# Effects of vitamin D supplementation on oxidative stress biomarkers of Iranian women with polycystic ovary syndrome: a meta-analysis study

**DOI:** 10.61622/rbgo/2024rbgo37

**Published:** 2024-06-27

**Authors:** Camila Maria Sampaio Ferreira Avelino, Rosângela Ferreira Frade de Araújo

**Affiliations:** 1 Laboratory of Immunopathology Keizo Asami Universidade Federal de Pernambuco Recife Brazil Laboratory of Immunopathology Keizo Asami, Universidade Federal de Pernambuco, Recife, PE, Brazil.; 2 Department of Biochemistry Universidade Federal de Pernambuco Recife Brazil Department of Biochemistry, Universidade Federal de Pernambuco, Recife, PE, Brazil.

**Keywords:** Polycystic ovary syndrome, Hyperandrogenism, Oxidative stress, Antioxidant therapy, Cholecalciferol

## Abstract

**Objective:**

To identify the impact of redox imbalance on the clinical evolution of patients with polycystic ovary syndrome and carry out a qualitative and quantitative projection of the benefits of vitamin D supplementation.

**Data sources:**

Combinations of the keywords *polycystic ovary syndrome, vitamin D, oxidative stress, reactive oxygen species, antioxidant*, and *free radicals* were used in PubMed, Cochrane Library, LILACS, EMBASE, and Web of Science databases. The last search was conducted on August 22, 2023.Selection of studies: Based on the inclusion and exclusion criteria, studies were selected considering a low risk of bias, published in the last 5 years in English, which investigated the effects of vitamin D supplementation in women with PCOS, focusing on oxidative stress markers. Of the 136 articles retrieved, 6 intervention studies (445 women) were included.

**Data collection:**

The risk of bias in included studies was assessed using the Jadad scale, and analysis and visualization of continuous data were performed using Review Manager 5.4.1, summarized as standardized mean differences (SMD) with confidence intervals (CI) of 95%.

**Data synthesis:**

Vitamin D effectively reduced malondialdehyde (*P*=0.002) and total testosterone (*P*=0.0004) levels and increased total antioxidant capacity levels (*P*=0.01). Although possible improvements in the modified Ferriman–Gallwey hirsutism score, levels of sex hormone-binding globulin, and free androgen index were identified and the results were not statistically significant.

**Conclusion:**

Vitamin D is a promising alternative for the treatment of PCOS with a positive influence on the oxidative, metabolic, and endocrine disorders of this syndrome.

## Introduction

Polycystic ovary syndrome (PCOS) is a common endocrinopathy affecting women of reproductive age and is associated with metabolic, reproductive, and endocrine complications. It is characterized by chronic anovulation, hyperandrogenism, and polycystic ovaries as well as an increased risk of hyperinsulinemia, insulin resistance (IR), type 2 diabetes mellitus (DM2), and metabolic syndrome.^(1,2)^

Although extensive clinical data have been published, a combination of “polycystic ovary syndrome” and “pathogenesis” in Medline yields 11,095 results, but several aspects remain unclear. Some hypotheses support the pathophysiology of PCOS, including disturbances in the hypothalamic-pituitary-ovarian axis and gonadotropin action, increased inflammation, and oxidative stress (OS). Revealed when the levels of oxidants significantly outweigh the antioxidant defenses, OS is associated with hyperandrogenism, IR, and metabolic dysfunction in PCOS. Therefore, understanding the mechanisms underlying the oxidative state in PCOS is important for the treatment and prevention of unfavorable prognosis.^(3-7)^

Along with known therapies, such as gonadotropins and insulin sensitizers, the role of antioxidants in PCOS, especially vitamin D supplementation, has been investigated. In studies by Łagowska et al.^(8)^and Miao et al.^9^ vitamin D has been shown to improve the clinical conditions secondary to PCOS, including IR, hyperandrogenism, and glycolytic metabolism. The beneficial effects of vitamin D have also been observed in reproductive health as its receptor is expressed in the ovary, endometrium, and myometrium, and its activation can regulate the expression of numerous genes. Furthermore, research suggests that vitamin D deficiency (DVD) is common in PCOS and may be related to metabolic and endocrine disorders, being a beneficial and promising therapy for the syndrome.^(8-11)^

Literature on the association among low vitamin D levels, OS, and PCOS is scarce. Thus, this meta-analysis aimed to update the role of vitamin D in the pathogenesis of PCOS, focusing on OS biomarkers, and demonstrating evidence that encourages further intervention studies.

## Methods

### Protocol and Registration

The present study was designed and reported according to the Preferred Reporting Items for Systematic Reviews and Meta-Analyses (PRISMA),^(12)^through the analysis of scientific articles that answer the following guiding question: “Does vitamin D supplementation offer a simple, low-cost strategy to improve antioxidant status and consequent quality of life in PCOS?” The study protocol was registered in the PROSPERO International Prospective Register of Systematic Reviews (registration number: CRD42023456845).

### Information sources

The bibliographic search was carried out using electronic databases, namely MEDLINE/PubMed, LILACS, EMBASE, Web of Science, and the Cochrane Library using the following combinations of Medical Subject Heading (MeSH) terms, advanced search terms, and Boolean operators: “Polycystic Ovary Syndrome AND (Oxidative Stress OR Reactive Oxygen Species OR free radicals OR antioxidant) AND vitamin D”.

The final search was conducted on December 2, 2022, and updated on Agust 22, 2023. The eligibility assessment was performed independently and standardized by the consensus of the authors, by screening the titles and abstracts of all selected articles; when the abstracts did not provide the necessary information, the full text was revised. Included in this review were studies published in full text in the last 5 years, in the English language, focused on patients aged between 13-19+ years and who met the eligibility criteria.

### Eligibility criteria

The included studies met the following criteria: (1) women of reproductive age with PCOS undergoing a vitamin D protocol; (2) an association between the serum levels of vitamin D and OS; and (3) comparative studies containing randomized clinical trials and case-control studies. A diagnosis of PCOS was considered valid if it met the Rotterdam (2003) or NIH (1990) criteria. The exclusion criteria were incomplete studies (without adequate diagnostic information or data available for analysis), studies not relevant to the assessment of vitamin D treatment in PCOS, studies with a high risk of bias, study designs, such as case reports, reviews, letters, meta-analyses, comments, editorials, protocols, and animal experiments.

After selecting the studies, descriptive information from each one was extracted and summarized in a spreadsheet to standardize the results obtained. The data in the spreadsheet were: author, year of study, sample size, age and BMI of enrolled patients, intervention measures, trial duration, and outcomes of interest.

### Study risk of bias assessment

The quality of the studies was analyzed using the Jadad scale,^(13)^ with a maximum score of 5, and a high-quality study was defined by a threshold of at least 3 points. The risk of bias was assessed as high or low for randomization (randomized schedule generation and allocation sequence), blinding (group comparability and determination of the outcome of interest), and performance (loss and/or exclusion of participants and justifications). Each item contained a sequence of questions to be answered by the researchers; additionally, the aforementioned point classification system allowed a semi-quantitative analysis of the quality of the articles.^(13)^

The Review Manager 5.4.1 software was adopted for the analysis and visualization of the data. For continuous outcomes, standardized mean differences (SMD) and 95% confidence interval (CI) were derived for each study. Sample size per group (N), mean values, and standard deviations (SD) were also reported. When multiple doses of the treatment were administered, the group that received the highest dose was recorded. If the results were only presented graphically, the online tool Web Plot Digitizer was used for quantification.

## Results

### Literature Identification

**Figure 1:**
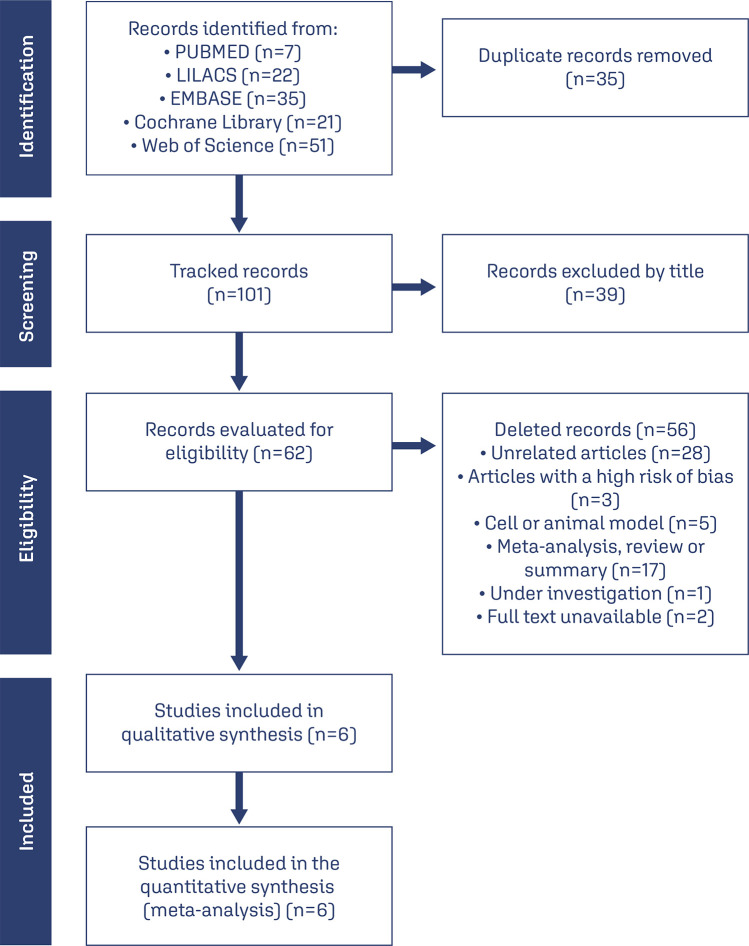
Study selection

In total, 136 potential articles were retrieved using the initial search strategy. After removing duplicates and reviewing the titles, 62 publications were carefully inspected for eligibility. Publications with insufficient data and/or those that did not meet the objectives of the systematic review or meta-analysis were excluded. Overall, six intervention studies met the inclusion criteria and were included in the final analysis (Figure 1).

### Study Characteristics

The data from the included intervention studies are summarized in chart 1. The studies found in the search that met the inclusion criteria were conducted in Asia and included only Iranian women. The year of publication ranged from 2017 to 2019. The sample sizes were heterogeneous across the studies, ranging from 60 to 105. The mean age of women with and without PCOS ranged from 18 to 28 years. The included patients did not use concomitant medications, except metformin, with an influence on the PCOS components before or during the studies. The Rotterdam criteria have been unanimously used to assess PCOS; however, none of the studies reported the time until menarche, which is essential to establish the diagnosis, as it can influence the assessment of symptoms, such as hirsutism, severe acne, and menstrual irregularities.^(14)^

**Chart 1:** Characteristics of included studies

Study, year	Sample size	Intervention	Relevant results*	Average age (years)		Average BMI (baseline)		Duration
				Experimental group	Control group	Experimental group	Control group	
Fatemi et al. (2017)^(15)^	105	Vitamin D3 (50,000 IU/once in two weeks) and vitamin E (400 mg/day), or placebo group;	1, 16	28 ± 4.2	28.1 ± 3.7	26.5 ± 2.9	26.1 ± 3.5	8 weeks
Jamilian et al. (2017)^(16)^	90	Vitamin D (1,000 or 4,000 IU/day) + metformin or vitamin D placebo + metformin;	1, 3, 5, 6, 7, 8, 11, 12, 13, 14	Low dose: 26 ± 5 High dose: 28 ± 5	25 ± 5	Low dose: 33 ± 5 High dose: 31 ± 6	30 ± 6	12 weeks
Maktabi et al. (2017)^(17)^	70	Vitamin D supplements (50,000 IU) every 2 weeks or placebo;	2, 8, 11, 12, 13	18-40	18-40	NA	NA	12 weeks
Jamilian et al. (2018)^(18)^	60	Vitamin D (50,000 IU/2x per week) + omega-3 fatty acids (2 g/day) or placebo;	2, 3, 5, 8, 15	26.8 ± 4.4	25.1 ± 3.7	27.4 ± 3.9	27.1 ± 7	12 weeks
Nasri et al. (2018)^(19)^	60	Vitamin D3 (1,000 IU/day) + EPO (1000 mg/day), or placebo;	1, 2, 4, 9, 10	26.4 ± 8.1	25.4 ± 4.7	26.4 ± 4.7	26.9 ± 4.9	12 weeks
Ostadmohammadi et al. (2019)^(20)^	60	Vitamin D (50,000 IU/every 2 weeks) + probiotic (8×10^9 CFU/day) or placebo;	2, 3, 4, 5, 8, 14, 15	24.4 ± 4.7	25.4 ± 5.1	24.3 ± 4.2	25.1 ± 4.9	12 weeks

*Relevant results: 1. Increase in 25-hydroxyvitamin D [25(OH)D]; 2. Reduction in malondialdehyde (MDA) levels; 3. Increased total antioxidant capacity (TAC); 4. Increased levels of reduced Glutathione (GSH); 5. Decreased total testosterone (TT) levels; 6. Reduction of the free androgen index (FAI); 7. Increased levels of sex hormone binding globulin (SHBG); 8. Reduction of C-reactive protein (CRP); 9. Reduction of Triglycerides (TG); 10. Reduction of High-Density Lipoprotein Cholesterol (HDL-C); 11. Reduction of Fasting Plasma Glycemia (FPG); 12. Reduction of serum insulin levels; 13. Improvement of evaluation of the homeostatic model of insulin resistance (HOMA-IR); 14. Improvement of hirsutism; 15. Improvement in mental health; 16. Improvement in ovulation rates or clinical pregnancy.

Over the past five years, interventional trials have investigated the potential effects of vitamin D on the metabolic, endocrine, and clinical parameters associated with OS in patients with PCOS. Two herbal medicines, an insulin sensitizer, and a probiotic, were investigated for co-administration with vitamin D. The supplementation period ranged from 8 to 12 weeks. Isolated vitamin D was investigated in one study,^(17)^ and its association with metformin, vitamin E, evening primrose oil, omega 3, and probiotics was investigated in single studies.^(15,16,18-20)^

### Risk of bias

**Figure 2:**
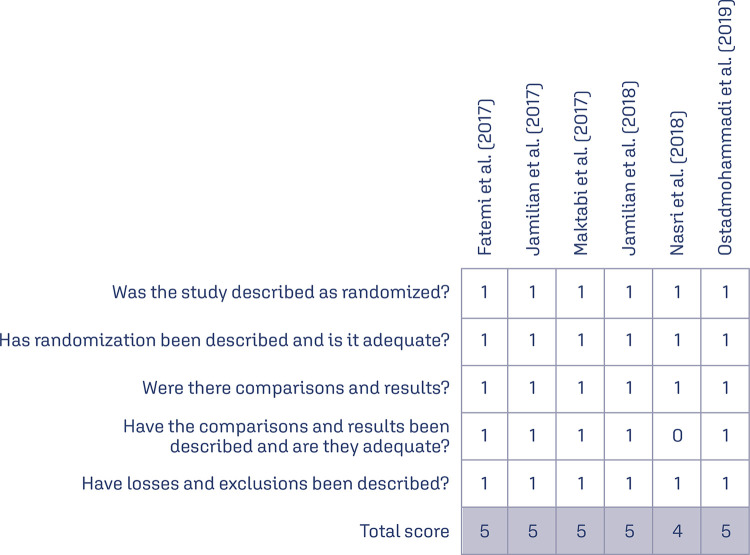
Summary of risk of bias

Included studies^(15-20)^in the present meta-analysis were assessed using the Jadad scale^(13)^ and had a low risk of bias for randomization, blinding, and performance. In general, clinical trials were conducted safely and presented adequate quality (Figure 2).

### Outcomes of vitamin D

**Figure 3:**
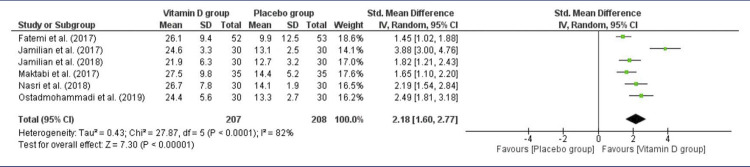
Vitamin D levels after intervention in PCOS patients

The vitamin D levels were evaluated in six studies with 415 women (207 in the intervention group and 208 in the Control Group), considering the high-dose group in Jamilian et al.^(16)^Using a random-effects model, the SMD was 2.18 (*P*<0.00001), demonstrating that vitamin D supplementation, alone or in combination, led to a significant increase in the 25(OH)D levels compared to the Control Group (Figure 3). There was significant heterogeneity among the studies [I^2^ = 82%, *P*<0.0001].

### Outcomes of oxidative stress markers

**Figure 4:**
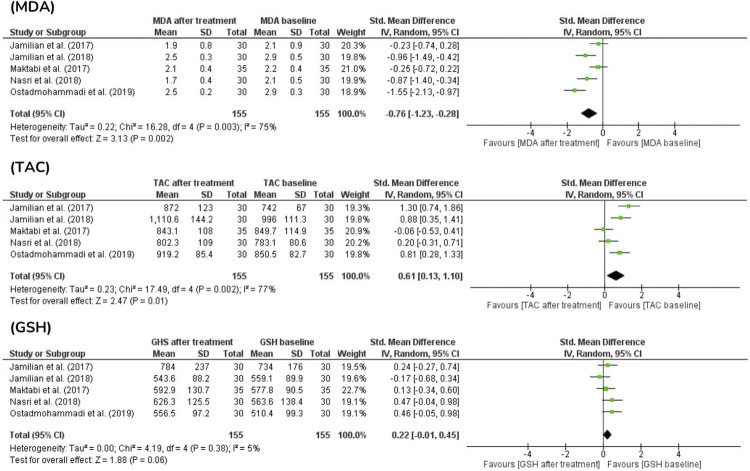
Oxidative stress markers before and after administration of vitamin D in PCOS patients

The malondialdehyde (MDA) and total antioxidant capacity (TAC) measurements of 310 patients were reported in five studies.^(16-20)^ After retrieving the data from the treatment group only (155 women) before and after the intervention, the results showed that vitamin D had antioxidant potential as it reduced the MDA levels (*P*=0.002) and increased the TAC levels (*P*=0.01) in the included patients. However, in two of five studies,^(16,17)^there was no statistically significant difference in the MDA level, and only one study,^(17)^showed a reduction in TAC at the end of the test. The glutathione (GSH) levels were analyzed in 310 patients in five studies.^(16-20)^The results showed that when comparing the baseline and post-supplementation values among the 155 patients in the treatment group, no statistically significant difference was observed in the effect of vitamin D on GSH (*P*=0.06). However, increased GSH levels were observed in 125 patients (Figure 4).

### Outcomes of androgens and hirsutism

**Figure 5:**
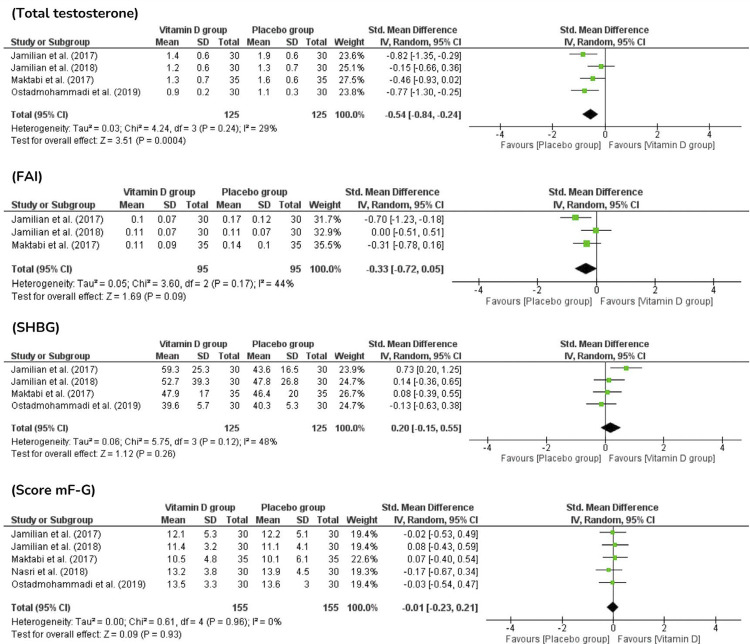
Androgen levels and hirsutism after administration of vitamin D in PCOS patients

The total testosterone (TT) level was measured and compared to the placebo, vitamin D significantly reduced the total testosterone levels (*P*=0.0004; four studies; 250 women). Two studies^(18,20)^ demonstrated an advantageous effect of antioxidant supplementation on this outcome. Compared with the placebo and after vitamin D treatment, the FAI index showed a decrease, sex hormone binding globulin (SHBG) levels showed improvement in three studies, and the modified Ferriman–Gallwey hirsutism (mF-G) score showed improvement. Despite the positive observations, the results were not statistically significant (*P*=0.09, 3 studies, 190 women; *P*=0.26, 4 studies, 125 women; *P*=0.93, 5 studies, 310 women, respectively). Particularly, vitamin D appears to benefit the clinical picture of patients, as hirsutism is present in most PCOS phenotypes (Figure 5).

## Discussion

Research has revealed a growing number of abnormalities in OS markers in patients with PCOS. As various syndrome associations can contribute to local and systemic sources, including hyperandrogenism, obesity, IR, and inflammation, redox imbalance has been suggested as a pathogenic inducer of PCOS. Owing to the close relationship between OS and PCOS, antioxidant agents have been investigated for symptom management beyond traditional strategies. Among them, the performance of vitamin D stands out, due to its positive role in the rescue of OS-induced damage to cells and tissues, and is a good therapeutic resource for PCOS.^(2,4,6,10,15-24)^

The main action of 25(OH)D is calcium homeostasis and bone metabolism, in addition to its role in cell differentiation and proliferation mediated by the vitamin D nuclear receptor (VDR), which regulates numerous genes. Studies have indicated that this active metabolite can also reduce OS by inhibiting the antiprotease activity and acting on erythroid nuclear factor 2-related factor 2 (Nrf2), which is considered the master regulator of the body’s antioxidant response. Additionally, vitamin D increases the proliferation of monocytes into macrophages, thereby controlling infection. This meta-analysis explored the effect of vitamin D on metabolic, hormonal, and clinical parameters in 445 patients with PCOS. The results suggest that intervention improved hirsutism and the hormonal profile, increased TAC, and presented an advantageous reduction in MDA. In contrast, no significant effect was observed on the GSH levels.^(10,11,22)^

MDA is the major end-product of lipid peroxidation, is highly toxic, and is negatively correlated with insulin sensitivity in PCOS. Among the six included intervention studies, five reported that antioxidant supplementation in women with PCOS decreased the serum MDA concentrations compared to the control groups. Maktabi et al.^(17)^evaluated the effects of isolated vitamin D in women with DVD and B-SOP phenotypes (hyperandrogenism and ovulatory dysfunction, without polycystic ovaries). The authors concluded that the intervention reduced the serum CRP and MDA concentrations; however, it did not influence other markers of inflammation and OS.^(2,17)^

MDA, an important marker of OS, was previously investigated by Foroozanfard et al.^(25)^ and was found to be significantly reduced after combined calcium and vitamin D therapy in overweight women, women with DVD, and women with PCOS. In another meta-analysis of PCOS in 1,481 women, a 47% increase in the MDA levels was observed, suggesting a redox imbalance in these patients. Previous studies have also shown that increased lipid and protein peroxidation in the follicular fluid can have a negative impact on the oocyte quality and fertilization rates in women with PCOS.^(21,25-28)^

Four studies reported a positive correlation between vitamin D therapy and plasma TAC levels. Responsible for measuring the antioxidant potential of body fluids, TAC refers to the ability of serum to eliminate free radical production and has been reported to be significantly low in various diseases, including PCOS. A meta-analysis of randomized clinical trials showed that women with PCOS showed decreased MDA and CRP levels and increased TAC levels after using vitamin D; however, similar to our findings, they did not have significant effects on GSH levels.^(2,6,29,30)^

Jamilian et al.^(18)^reported significant changes in TAC and MDA levels after vitamin D and omega-3 supplementation in women with PCOS. Similarly, Ostadmohammadi et al.^(20)^concluded that probiotics and vitamin D reduced the MDA levels and increased the TAC and GSH levels compared to the placebo. As a cofactor of various antioxidant enzymes, GSH plays a central role in protecting against damage induced by reactive oxygen species (ERO), acting as an endogenous antioxidant when there is a rupture of the redox homeostasis. OS can generate DNA damage, interrupt redox pathways, and cause cellular death, which is common in the development of pathologies linked to the female reproductive system. Thus, the reduction of GSH levels, combined with an increased oxidizing state, explain the mitochondrial dysfunction in women with PCOS.^(5,6,18,20,23)^

Similar findings were observed by Jamilian et al.,^(16)^ indicating that the beneficial effects of TAC are due to high-dose vitamin D treatment in women with IR and PCOS when compared to the low-dose and placebo groups. Furthermore, supplementation significantly reduced serum insulin, HOMA-IR, TT, FAI, and hirsutism and increased SHBG. The opposite conclusion that dysregulation of vitamin D metabolism may be a consequence of PCOS was presented by He et al.^(31)^A quantitative analysis of 3,182 participants, did not reveal changes in the metabolic and endocrine functions after antioxidant therapy, although this association has been observed in other cross-sectional and case-control studies. This discrepancy can be explained by the short study duration and the differences between basal vitamin D levels, insulin levels, and HOMA-IR scores of the included participants.^(16,31-33)^

In addition, Nasri et al.^(19)^and Ostadmohammadi et al.^(20)^ found significant increases in the GSH levels after antioxidant supplementation in patients with PCOS, as previously described by Foroozanfard et al.^(25)^ Because OS is associated with PCOS pathogenesis, improvements in the GSH levels and reduced ROS production and proinflammatory factors by vitamin D may explain its antioxidant effects.^(2,11,19,20,23,25)^

In contrast to the positive results cited, Fatemi et al.^(15)^found no changes in the oxidative factors after vitamin E and D3 supplementation in women with PCOS-related infertility. The intervention improved the vitamin D levels and increased clinical implantation and pregnancy rates. However, there was no reduction in the MDA or increase in TAC, although this was observed on the day of egg collection, on which OS had not yet been induced in patients. According to Agarwal et al.,^(34)^ovarian stimulation can influence ROS production and disturb the redox balance, leading to OS. Thus, the results are in accordance with the literature and indicate that the antioxidant dosage used was insufficient to overcome or reverse OS induced after *in vitro* fertilization.^(15,34)^

Other important parameters, including TT, FAI, SHBG, and hirsutism levels, were also investigated. Reported in approximately 75% of patients, hyperandrogenism is one of the main characteristics of PCOS and is clinically characterized by signs, such as hirsutism, acne, and androgenic alopecia. Testosterone, SHBG, and FAI are diagnostic markers of biochemical hyperandrogenism, and inverse associations between these markers and serum 25(OH)D levels have been reported in some studies.^(1,31-33,35)^

In this meta-analysis, vitamin D showed an apparent improvement in the hormonal profile of the included patients, as TT and FAI indicators decreased after the intervention, and SHBG levels increased, suggesting that the mF-G score can be improved by antioxidant treatment in PCOS. Considering the gold standard for the clinical assessment of hirsutism, the mF-G score evaluates the hair growth in nine androgen-sensitive body areas, with scores ranging from no terminal hair growth (equal to 0) to hirsutism (equal to 8).^(35)^However, considering these findings, only TT analysis yielded statistically significant results (Figure 5).

Interestingly, the only population investigated by the 6 studies included in our review was the Iranian population. According to Jalilian et al.,^(36)^who investigated complications associated with PCOS in this population, hirsutism was reported in 25-30% of women, while the prevalence of acne was 26%. Increased levels of androgens are present in approximately 80% of women with severe acne, thus having a significant relationship with the incidence of PCOS. This reinforces our findings on the hormonal profile of the women included. Furthermore, the prevalence of PCOS in Iran varies between 7% and 15% and the risk of PCOS was higher in the presence of food insecurity and low economic levels and negatively associated with physical exercise and healthy eating patterns in Iranian women with PCOS. Furthermore, results from another study showed that the frequency of metabolic syndrome was higher in the group of Iranian women with PCOS compared to the control group, indicating that hyperandrogenic components are predictors of metabolic disorders in PCOS.^(37-39)^

The high heterogeneity present in this meta-analysis can be explained by the diversity in intervention doses and populations studied, since some studies analyzed normal ovulatory non-infertile women and others, specifically analyzed infertile women, and the B-PCOS phenotype, leading to conflicting results. In addition, individual vitamin D levels may vary owing to the influence of diet and sun exposure, variations not considered in transverse studies, limited by the nature of evaluating a single point in time.^(22)^The other aspects to be considered are the variations between studies regarding the sample size and methodology as well as a limited number of reviewed studies.

In clinical applications, vitamin D significantly influenced the MDA, TAC, and TT levels there by improving the OS and hyperandrogenism. However, the potential underlying mechanisms of vitamin D in PCOS, adaptable duration of treatment, appropriate dosages, and nature of the association (causality) between serum vitamin D and OS have not yet been fully delineated. There is a need for studies with diverse populations (women with and without PCOS, with different nationalities, and PCOS phenotypes), greater sampling, and evaluation of other oxidative markers aimed at a better understanding of the effect of vitamin D on the damage caused by OS in PCOS.

## Conclusion

Literature on the association between vitamin D status and redox imbalance in polycystic ovary syndrome is limited and has provided conflicting results. In the present meta-analysis, although we only found studies including Iranian women, the effectiveness of antioxidant interventions in the rescue of oxidative balance in PCOS was demonstrated in this group. The OS plays a significant role in female reproduction and is related to hyperandrogenism, obesity, and IR in PCOS patients. In addition to its significant effects on MDA and TAC, vitamin D positively influenced the serum levels of TT. Therefore, it is a promising alternative for the treatment of PCOS and metabolic and endocrine disorders caused by this syndrome.
